# Integrated multiple microarray studies by robust rank aggregation to identify immune-associated biomarkers in Crohn's disease based on three machine learning methods

**DOI:** 10.1038/s41598-022-26345-1

**Published:** 2023-02-15

**Authors:** Zi-An Chen, Hui-hui Ma, Yan Wang, Hui Tian, Jian-wei Mi, Dong-Mei Yao, Chuan-Jie Yang

**Affiliations:** 1 Department of Gastroenterology, The Second Hospital of Hebei Medical University, Shijiazhuang, 050000 Hebei China; 2 Hebei Key Laboratory of Gastroenterology, Hebei Institute of Gastroenterology, Hebei Clinical Research Center for Digestive Disease, Shijiazhuang, 050000 Hebei China

**Keywords:** Crohn's disease, Diagnostic markers

## Abstract

Crohn's disease (CD) is a complex autoimmune disorder presumed to be driven by complex interactions of genetic, immune, microbial and even environmental factors. Intrinsic molecular mechanisms in CD, however, remain poorly understood. The identification of novel biomarkers in CD cases based on larger samples through machine learning approaches may inform the diagnosis and treatment of diseases. A comprehensive analysis was conducted on all CD datasets of Gene Expression Omnibus (GEO); our team then used the robust rank aggregation (RRA) method to identify differentially expressed genes (DEGs) between controls and CD patients. PPI (protein‒protein interaction) network and functional enrichment analyses were performed to investigate the potential functions of the DEGs, with molecular complex detection (MCODE) identifying some important functional modules from the PPI network. Three machine learning algorithms, support vector machine-recursive feature elimination (SVM-RFE), random forest (RF), and least absolute shrinkage and selection operator (LASSO), were applied to determine characteristic genes, which were verified by ROC curve analysis and immunohistochemistry (IHC) using clinical samples. Univariable and multivariable logistic regression were used to establish a machine learning score for diagnosis. Single-sample GSEA (ssGSEA) was performed to examine the correlation between immune infiltration and biomarkers. In total, 5 datasets met the inclusion criteria: GSE75214, GSE95095, GSE126124, GSE179285, and GSE186582. Based on RRA integrated analysis, 203 significant DEGs were identified (120 upregulated genes and 83 downregulated genes), and MCODE revealed some important functional modules in the PPI network. Machine learning identified LCN2, REG1A, AQP9, CCL2, GIP, PROK2, DEFA5, CXCL9, and NAMPT; AQP9, PROK2, LCN2, and NAMPT were further verified by ROC curves and IHC in the external cohort. The final machine learning score was defined as [Expression level of AQP9 × (2.644)] + [Expression level of LCN2 × (0.958)] + [Expression level of NAMPT × (1.115)]. ssGSEA showed markedly elevated levels of dendritic cells and innate immune cells, such as macrophages and NK cells, in CD, consistent with the gene enrichment results that the DEGs are mainly involved in the IL-17 signaling pathway and humoral immune response. The selected biomarkers analyzed by the RRA method and machine learning are highly reliable. These findings improve our understanding of the molecular mechanisms of CD pathogenesis.

## Introduction

Crohn's disease (CD) is a complex genetic disorder likely caused by genetic, microbial, environmental, and immune factors^1–3^, with chronic diarrhea, abdominal pain, and weight loss being among the most common symptoms. Currently, there is no curative medical approach for CD^4^. Therefore, the importance of understanding the cellular and molecular mechanisms involved in CD pathogenesis, exploring novel intervention targets, and identifying potential biomarkers as diagnostic indicators cannot be overlooked.

Several immune-associated cell types present in the intestinal mucosa are reported to contribute to CD pathophysiology, including dendritic cells^5–7^ and lymphocytes^8,9^. However, the inherent complexity of CD, as manifested by a widely variable clinical course, makes it difficult to dissect disease mechanisms and to identify biomarkers that play a key role in disease progression. With the development of genomic sequencing technology, an increasing number of microarray datasets have been reported, providing an ideal source to investigate various molecular roles in CD. In recent years, several studies utilizing microarray technology have been published to identify CD biomarkers^10–12^. However, as these studies included relatively small sample sizes or lacked verification in external datasets, representing the molecular characteristics of this complex disease is difficult. The identification of novel biomarkers in CD cases based on larger samples through machine learning approaches may inform the diagnosis and treatment of diseases.

In this study, we constructed a CD cohort with the largest sample size to date through the robust rank aggregation (RRA) method^13^. A network of protein‒protein interactions (PPIs) was then built, and several functional modules were detected after identifying differentially expressed genes (DEGs). In addition, three machine learning algorithms, namely, support vector machine-recursive feature elimination (SVM-RFE), random forest (RF), and least absolute shrinkage and selection operator (LASSO), were applied to determine characteristic genes among multiple CD cohorts. We further illustrate the immune molecule-related functions in CD. This work reveals the key role of different immune molecules in the occurrence and development of CD.

## Materials and methods

### Search strategy for CD microarray datasets

**Figure 1:**
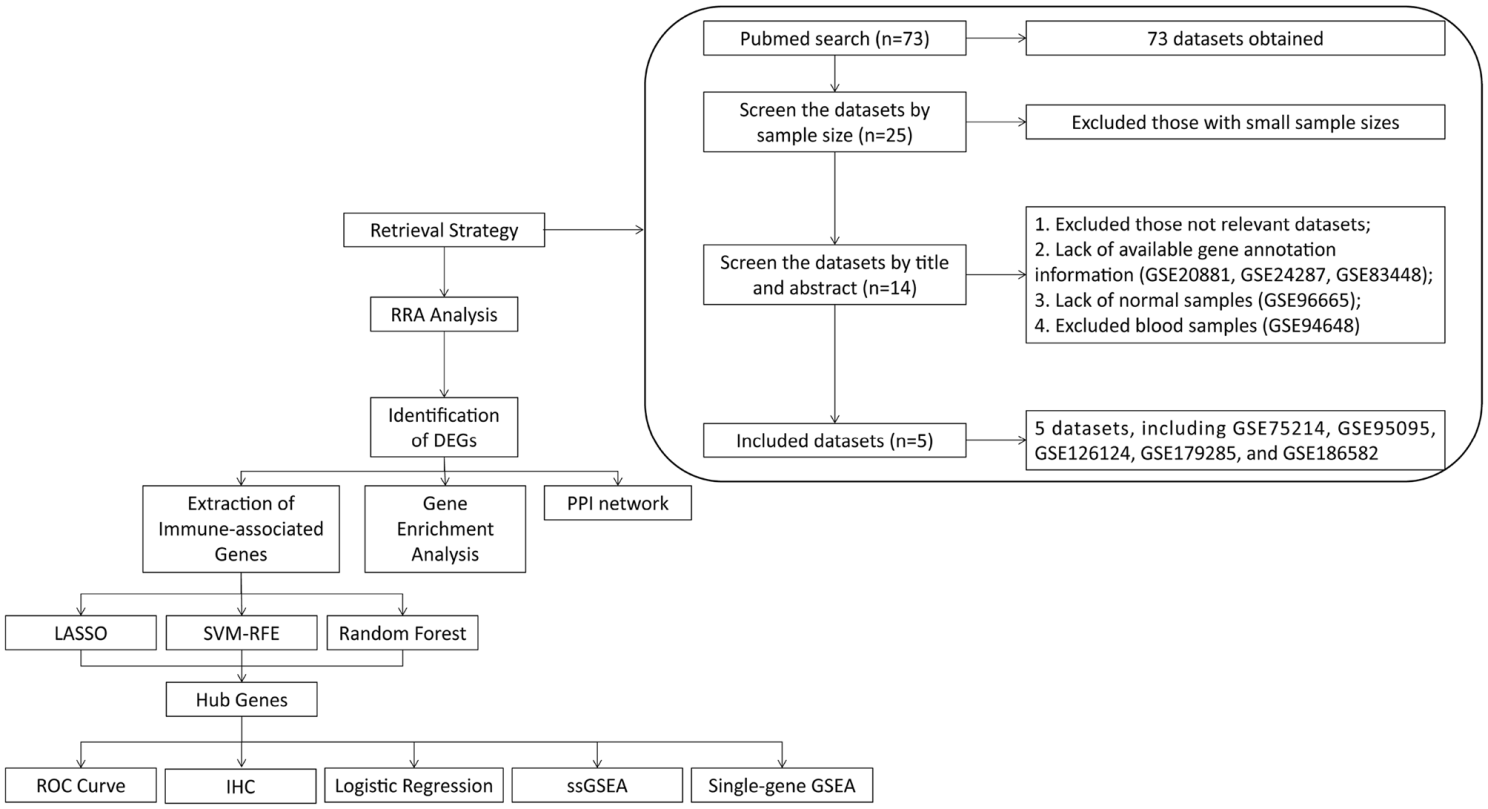
Flowchart of the overall study design.

A total of 73 datasets were collected from the Gene Expression Omnibus (GEO) database (https://www.ncbi.nlm.nih.gov/geo/) by systematic retrieval using the following keywords: ("Crohn Disease"[MeSH Terms] OR Crohn Disease [All Fields]) AND "Homo sapiens"[porgn] AND ("Expression profiling by array"[Filter] AND (“2012/01/01”[PDAT]: “2022/01/01”[PDAT])). Inclusion criteria were (1) sample size > 50, (2) both cases and controls included, (3) "ileum/colon" as sample source, and (4) available gene annotation information (listed in Fig. 1).

### Identification of DEGs in each CD dataset

The gene expression profiles of all datasets in the final analysis were downloaded from the GEO database (https://www.ncbi.nlm.nih.gov/geo/). For multiple probes targeting the same gene, the scaled expression values were averaged. The matrix file was extracted using PERL software (PERL version 5.6.1), and quantile normalization was performed using the normalizeBetweenArrays function^14^ in R software (R version 4.2.0). Due to the large fluctuations of the values in GSE179285, all values in these data were logarithmically transformed (e.g., "a" to "log(a + 1)") to make them conform to the requirements of a normal distribution and facilitate downstream data analysis. LogFC (log fold change) > 0.7 and adjusted P < 0.05 were set as the criteria for identifying DEGs.

### RRA analysis and identification of DEGs in the integrated cohort

Using RRA, all genes were sorted for each dataset and ranked based on their logFC with the limma package. The DEGs were then ranked using the ranked list and aggregated using the "RobustRankAggreg" package^13^ of R software. In this method, an adjusted P value determines the likelihood that DEGs will be identified in datasets with highly ranked genes. LogFC (log fold change) > 0.7 and adjusted P < 0.05 were set as the criteria for identifying DEGs.

### Functional and pathway enrichment analyses

We performed Gene Ontology biological process (GO-BP) analysis and Kyoto Encyclopedia of Genes and Genomes (KEGG) analysis^15–17^ on the DEGs identified by RRA using the limma and clusterProfiler packages. For enrichment analysis of DEGs, adjusted P < 0.05 was used^18^.

### PPI (protein‒protein interactions) network analysis

For the DEGs obtained by RRA analysis, a PPI network was constructed using the STRING website (https://cn.string-db.org/) with a parameter of confidence > 0.4. Visualization of the PPI network was performed by Cytoscape (Cytoscape version 3.7.2), and molecular complex detection (MCODE) (a plug-in in Cytoscape) was used to identify functional modules^19^.

### Analysis of CD- and immune-associated genes

A list of 2483 immune-associated genes was obtained from the Immunology Database and Analysis Portal (https://www.immport.org/shared/genelists). Candidate genes were determined by the intersection of DEGs and immune-related genes, followed by the elimination of gene symbols that do not exist in the five RRA analysis-associated datasets.

### Feature selection of characteristic biomarkers via three machine learning methods

We used LASSO^20^, SVM-RFE^21^, and RF^22^ to perform feature selection for diagnostic biomarkers for CD. The LASSO algorithm was applied with a turning/penalty parameter using tenfold cross-validation via the glmnet package^23^. With SVM-RFE, relevant characteristics are selected, and redundant characteristics are removed more effectively than with linear discriminant analysis or the mean squared error method. By using tenfold cross-validation, SVM-RFE was applied for feature selection, and the top 10 genes were determined as characteristic genes^24^. The RF algorithm is a randomization algorithm to reduce overfitting of a single decision tree and promote model accuracy based on numerous relevant decision trees from one training set; the top 10 genes were determined as the characteristic genes^25^.

For every dataset included in RRA, the genes obtained from the intersection of genes *selected* by the three methods were identified as characteristic genes. The area under the receiver operating characteristic (ROC) curve (AUC) was used to estimate diagnostic efficacy.

### Batch correction, data merging and PCA dimensionality reduction

With the combat function by the sva package, we reduced the batch differences and merged 3 cohorts: GSE75214 (GPL6244, Affymetrix), GSE126124 (GPL6244, Affymetrix), and GSE186582 (GPL570, Affymetrix) from the same company platform. The dataset was named the combined dataset. A principal component analysis (PCA) was performed to evaluate the magnitude of batch differences before and after correction.

### Construction of the diagnostic machine learning score

Univariate logistic regression analysis was performed to identify diagnostic genes in combined dataset patients (p < 0.05). The identified genes were further included in a multivariate logistic regression analysis to construct a potential machine learning score in CD. Finally, a formula for the risk score was established, and we calculated the risk score of each case as follows:

$${\text{RiskScore}} = \sum\limits_{t = 1}^{{\text{n}}} {{\text{Coefi}} \times {\text{Xi}}}$$

Coefi indicates the correlation coefficient of each gene, and X indicates the level of gene expression.

### Landscape of immune cell infiltration

To combine the datasets, the limma package and the combat function of the sva package^26^ were applied to preprocess and remove the batch effects of these three datasets. Based on the expression profiles of 29 immunity-relevant signatures, the single-sample gene set enrichment analysis (ssGSEA) method was utilized to determine the degree of immune cell infiltration.

### Gene set enrichment analysis

GSEA was performed on characteristic genes to elucidate their biological significance^27^. To achieve a normalized enrichment score for each analysis, 1000 gene set permutations were conducted. A false discovery rate (FDR) < 0.05 was regarded as significant enrichment to identify significant KEGG pathways.

### Verification of CD-associated characteristic genes by immunohistochemical (IHC) staining

To validate the results of genetic analysis at the transcriptional level, 6 patients with chronic colitis or CD were consecutively recruited between March and May 2021 in the Department of Gastroenterology at the Second Hospital of Hebei Medical University. Written informed consent was obtained from all individuals. In addition, the study was approved by the Ethics Committee of Second Hospital of Hebei Medical University. All research was performed in accordance with relevant guidelines/regulations. A total of 3 chronic colitis samples and 3 CD samples from human intestinal mucosal tissues were collected to perform histopathologic diagnosis by two pathologists.

The collected intestinal mucosa samples were fixed with 4% PFA and embedded in paraffin. IHC staining was performed as previously described^28^. Antibodies against the following were used: AQP9 (A8540; 1:200 dilution) and PROK2 (A6705; 1:200 dilution). HRP-labeled goat anti-rabbit antibody (AS014; 1:200 dilution, all from ABclonal, Wuhan, China) was used as the secondary antibody.

### Statistical analysis

All statistical tests were implemented using R software 4.1.3. The Wilcoxon test was applied to analyze the significant difference between two groups, and Spearman's correlation test was used to determine the correlation between the variables. A statistically significant P value was regarded as 0.05.

## Results

### Characteristics of the microarrays included in RRA analysis

The flow diagram of the CD dataset search strategy and inclusion and exclusion criteria is described in Fig. 1. According to the criteria described in the methods, a total of five datasets were included in further analysis: GSE75214^29^, GSE95095, GSE126124^30^, GSE179285^31^, and GSE186582^32^. Among these five datasets, 671 CD cases (including 308 inflamed samples of CD) were enrolled in the CD group, and 109 were enrolled in the control group. An overview of the microarray datasets included in the study is shown in Table 1.

**Table 1:** Characteristics of the included microarray datasets.

GSE ID	Participants (control/CD)	Analysis Type	Platform	Year	Tissues	Links
GSE75214	22/75	Array	GPL6244	2017	Colon/Ileum	https://www.ncbi.nlm.nih.gov/geo/query/acc.cgi?acc=GSE75214
GSE95095	12/48	Array	GPL14951	2019	Colon/Ileum	https://www.ncbi.nlm.nih.gov/geo/query/acc.cgi?acc=GSE95095
GSE126124	19/37	Array	GPL6244	2019	Colon	https://www.ncbi.nlm.nih.gov/geo/query/acc.cgi?acc=GSE126124
GSE179285	31/168	Array	GPL6480	2021	Colon/Ileum	https://www.ncbi.nlm.nih.gov/geo/query/acc.cgi?acc=GSE179285
GSE186582	25/343	Array	GPL570	2021	Ileum	https://www.ncbi.nlm.nih.gov/geo/query/acc.cgi?acc=GSE186582

### RRA integrated analysis

**Figure 2:**
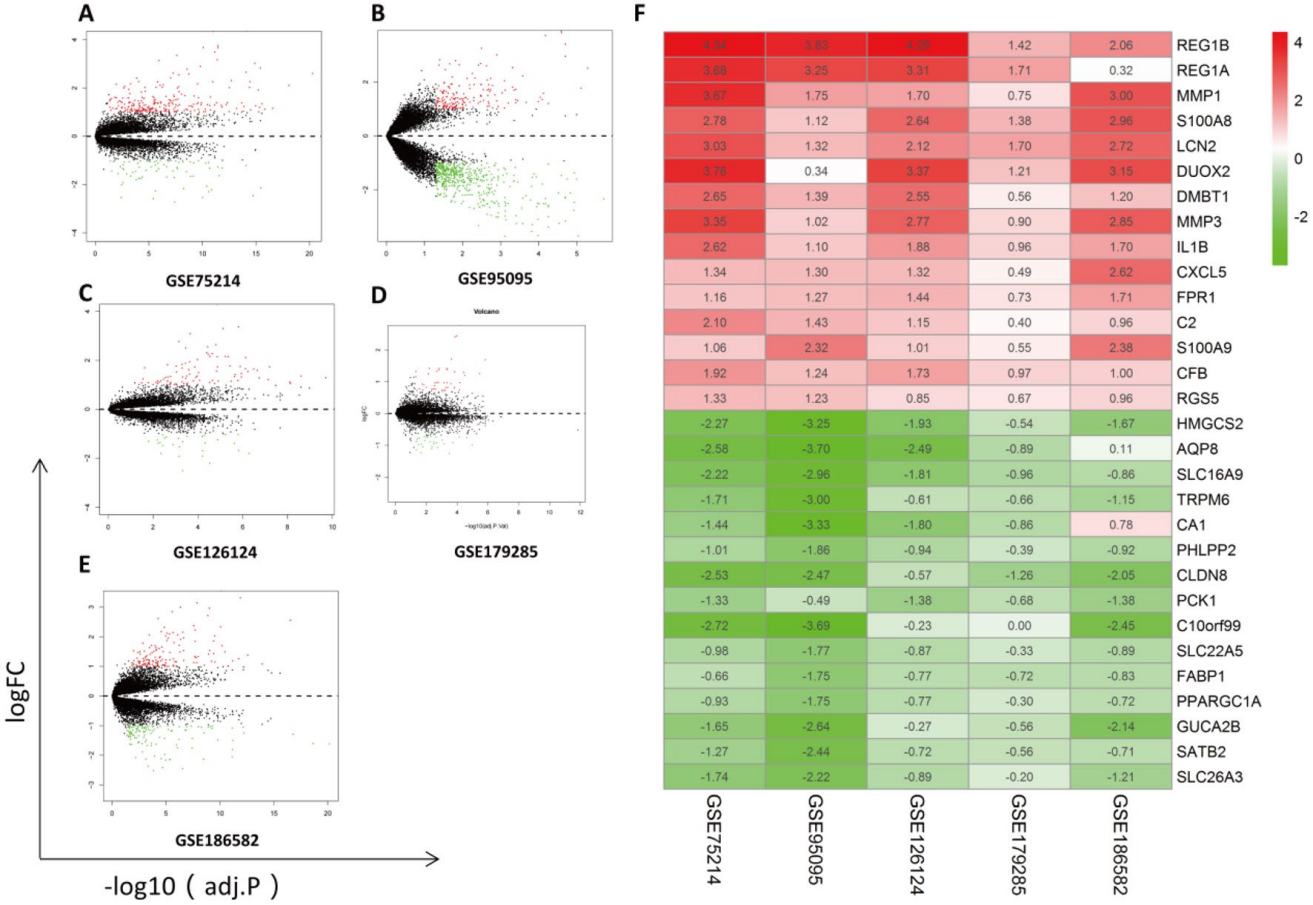
Identification of DEGs from a single dataset and RRA analysis. Volcano plots of DEG distributions in GSE75214 (**A**), GSE95095 (**B**), GSE126124 (**C**), GSE179285 (**D**), and GSE186582 (**E**). Upregulated genes are marked by red points, and downregulated genes are marked by green points; genes with no significant differences are marked by black points. (**F**) Heatmap of the top 15 DEGs (upregulated or downregulated) identified in RRA.

Before RRA analysis, all included cohorts were standardized to reduce batch differences among multicenter data (displayed in Supplementary Fig. 1). DEGs in each dataset were identified; volcano maps are illustrated in Fig. 2A–E.

After the RRA method, a total of 203 DEGs (83 downregulated and 120 upregulated) were identified. A heatmap including the top 15 DEGs (upregulated or downregulated) is illustrated in Fig. 2F. According to the analysis, the top 10 significant genes expressed in CD included five upregulated genes [REG1B (P = 2.76E−11), REG1A (P = 7.43E−10), MMP1 (P = 3.60E−09), S100A8 (P = 3.39E−08), and LCN2 (P = 7.78E−08)] and five downregulated genes [HMGCS2 (P = 3.60E−08), AQP8 (P = 1.55E−07), SLC16A9 (P = 1.55E−07), TRPM6 (P = 8.21E−07), and CA1 (P = 9.62E−07)]. Interestingly, the top 10 significant genes were somewhat similar to another RRA analysis from our previous study in ulcerative colitis (UC) (five upregulated genes: DUOX2, SLC6A14, MMP3, REG1A, and REG1B; and five downregulated genes: AQP8, HMGCS2, PCK1, SLC26A2, and ABCG2), indicating high homogeneity in clinical inflammatory bowel disease (IBD)^33^. Supplementary Table 1 lists the overall RRA results.

### DEG-based functional enrichment analysis

**Figure 3:**
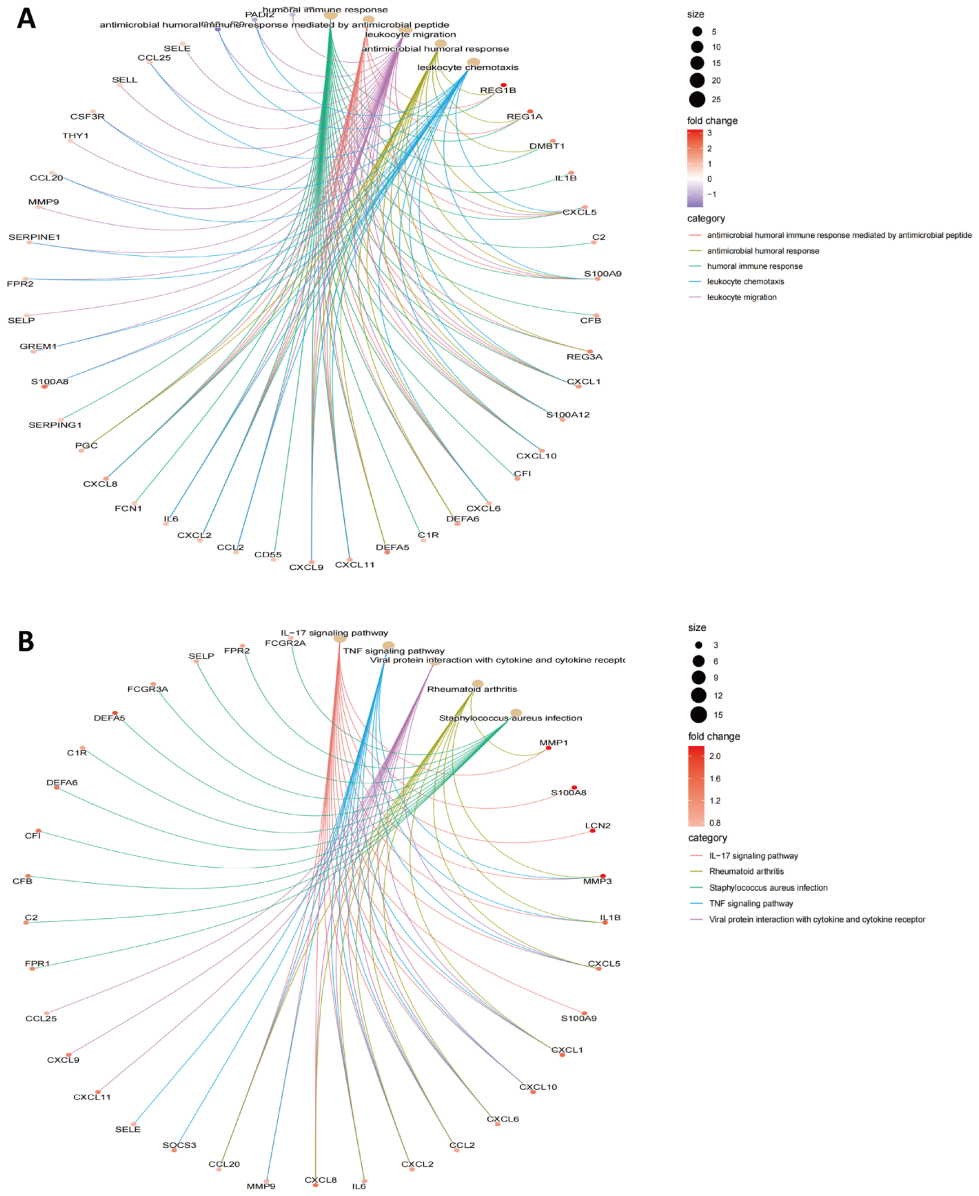
Functional enrichment analysis of DEGs. (**A**) GO-BP analysis and (**B**) KEGG pathway analysis.

The DEGs, including 120 upregulated and 83 downregulated genes, were subjected to GO-BP analysis and KEGG analysis, and the top five results are listed in Fig. 3A,B. The results showed humoral immune response, antimicrobial humoral immune response mediated by antimicrobial peptide, leukocyte migration, antimicrobial humoral response, and leukocyte chemotaxis to be the top five enriched BPs. The IL-17 signaling pathway, the TNF signaling pathway, viral protein interaction with cytokine and cytokine receptor, rheumatoid arthritis, and *Staphylococcus aureus* infection were found to be the top five enriched KEGG pathways (Fig. 3A). The detailed results are listed in Supplementary Table 2. Summarizing the results of our gene enrichment analysis and those in similar studies, we found that the results of KEGG analysis were similar to those of previously reported articles, i.e., IL-17 and TNF-α were significantly enriched in CD^11,34^; however, in BP analysis, our study found that a variety of humoral immunities, especially microbial-associated humoral immunity, were significantly enriched in CD, which has not been previously reported in the literature.

### PPI network analysis and identification of characteristic genes

**Figure 4:**
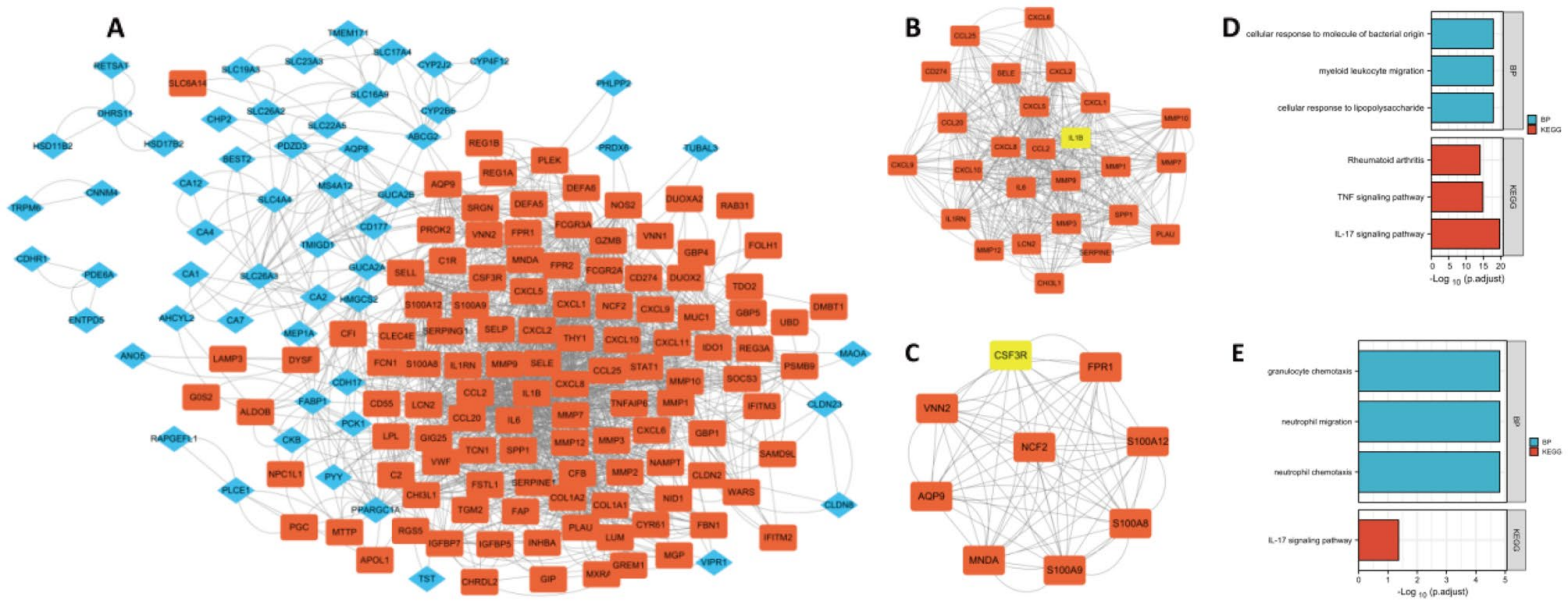
Visualization and module identification of the PPI network. (**A**) A total of 203 DEGs were mapped using Cytoscape software. Two PPI network modules were identified using the MCODE plug-in. (**B**) Module 1 comprised MMP9, CCL2, SERPINE1, MMP10, PLAU, MMP7, CCL25, MMP12, CD274, CHI3L1, LCN2, SPP1, MMP1, SELE, MMP3, CXCL5, CXCL6, CXCL9, CCL20, CXCL2, CXCL1, CXCL10, IL6, CXCL8, and IL1RN, with the seed gene IL1β. (**C**) Module 2 comprised S100A9, NCF2, FPR1, MNDA, AQP9, S100A12, VNN2, and S100A8, with the seed gene CSF3R. In functional enrichment analyses of the genes in module 1 (**D**) and module 2 (**E**), the red points represent upregulated genes, and the blue points represent downregulated genes.

Using the STRING website, a visual network of PPIs based on RRA analysis was constructed, including 203 nodes and 940 edges. The network was then imported into Cytoscape software for further analysis, with upregulated genes shown with orange markers and downregulated genes with blue markers (Fig. 4A). The top two modules with the highest scores were determined by MCODE. Module 1 comprised MMP9, CCL2, SERPINE1, MMP10, PLAU, MMP7, CCL25, MMP12, CD274, CHI3L1, LCN2, SPP1, MMP1, SELE, MMP3, CXCL5, CXCL6, CXCL9, CCL20, CXCL2, CXCL1, CXCL10, IL6, CXCL8, and IL1RN, with the seed gene IL1β (Fig. 4B). Module 2 comprised S100A9, NCF2, FPR1, MNDA, AQP9, S100A12, VNN2, and S100A8, with the seed gene CSF3R (Fig. 4C). The detailed scores of each module are shown in Supplementary Table 3.

Based on GO-BP enrichment analysis of module 1, the genes are mainly involved in cellular response to lipopolysaccharide, myeloid leukocyte migration, and cellular response to molecules of bacterial origin. KEGG analysis showed that these genes play a major role in the IL-17 signaling pathway, the TNF signaling pathway, and rheumatoid arthritis (Fig. 4D).

GO enrichment analysis of module 2 revealed the DEGs to be mainly related to neutrophil chemotaxis, neutrophil migration, and granulocyte chemotaxis. KEGG analysis revealed that these genes are mainly involved in the IL-17 signaling pathway (Fig. 4E).

### Determination of characteristic genes

Considering that the immune response is an important factor leading to the occurrence, progression and prognosis of CD, we next extracted immune-related genes among the DEGs, eliminated those not present in the five datasets, and obtained a total of 46 candidate immune genes (Supplementary Table 4).

In the following investigation, three different machine learning methods (LASSO, SVM-RFE and RF) were employed for *feature selection* and to determine characteristic genes in each dataset. As described above, when a gene was selected by the three methods at the same time, the gene was identified as a characteristic gene in each dataset.

The results were as follows. For GSE75214, CXCL1, STAT1, CXCL6, AQP9, LCN2, REG1A, GIP, and VIPR1 were selected by LASSO; LCN2, MMP12, VIPR1, REG1A, CCL20, FPR2, AQP9, IL1β, PYY, and CXCL2 were selected by SVM-RFE; and NOS2, IDO1, DMBT1, STAT1, AQP9, CXCL1, REG1A, LCN2, SOCS3, and CXCL6 were selected by RF. For GSE95095, PYY, SOCS3, CXCL1, SPP1, PROK2, DEFA5, CXCL10, LCN2, REG1A, PLAU, GIP, CCL2, and VIPR1 were selected by LASSO; CCL2, LCN2, VIPR1, PROK2, DEFA5, PYY, DES, CXCL6, GIP, and CXCL2 were selected by SVM-RFE; and CCL25, S100A12, DEFA5, SOCS3, SERPINA3, PROK2, GIP, CCL2, CXCL6, and PLAU were selected by RF. For GSE126124, STAT1, PROK2, LCN2, REG1A, CXCL11, and CXCL9 were selected by LASSO; IDO1, MMP12, PROK2, GREM1, CXCL5, AQP9, CXCL9, S100A8, LCN2, and CHP2 were selected by SVM-RFE; and AQP9, CXCL6, S100A8, IDO1, CSF3R, CXCL11, CXCL9, LCN2, CXCL1, and CXCL5 were selected by RF. For GSE179285, FPR1, SPP1, NOS2, CXCL10, GREM1, CHP2, LCN2, GIP, DES, CCL20, DMBT1, and VIPR1 were selected by LASSO; PROK2, CXCL10, LCN2, VIPR1, SPP1, FPR1, CSF3R, SERPINA3, S100A9, and DES were selected by SVM-RFE; and DEFA5, DEFA6, REG1A, LCN2, PROK2, CXCL1, SERPINA3, DUOX2, NOS2, and S100A8 were selected by RF. For GSE186582, PYY, CSF3R, SPP1, STAT1, NOS2, GREM1, LCN2, NAMPT, REG1A, GIP, DES, CCL2, and MMP9 were selected by LASSO; NAMPT, GIP, STAT1, LCN2, PROK2, SPP1, CXCL1, GZMB, PLAU, and PYY were selected by SVM-RFE; and NAMPT, GIP, STAT1, LCN2, PROK2, SPP1, CXCL1, GZMB, PLAU, and PYY were selected by RF. These results are detailed in Supplementary Table 5.

**Figure 5:**
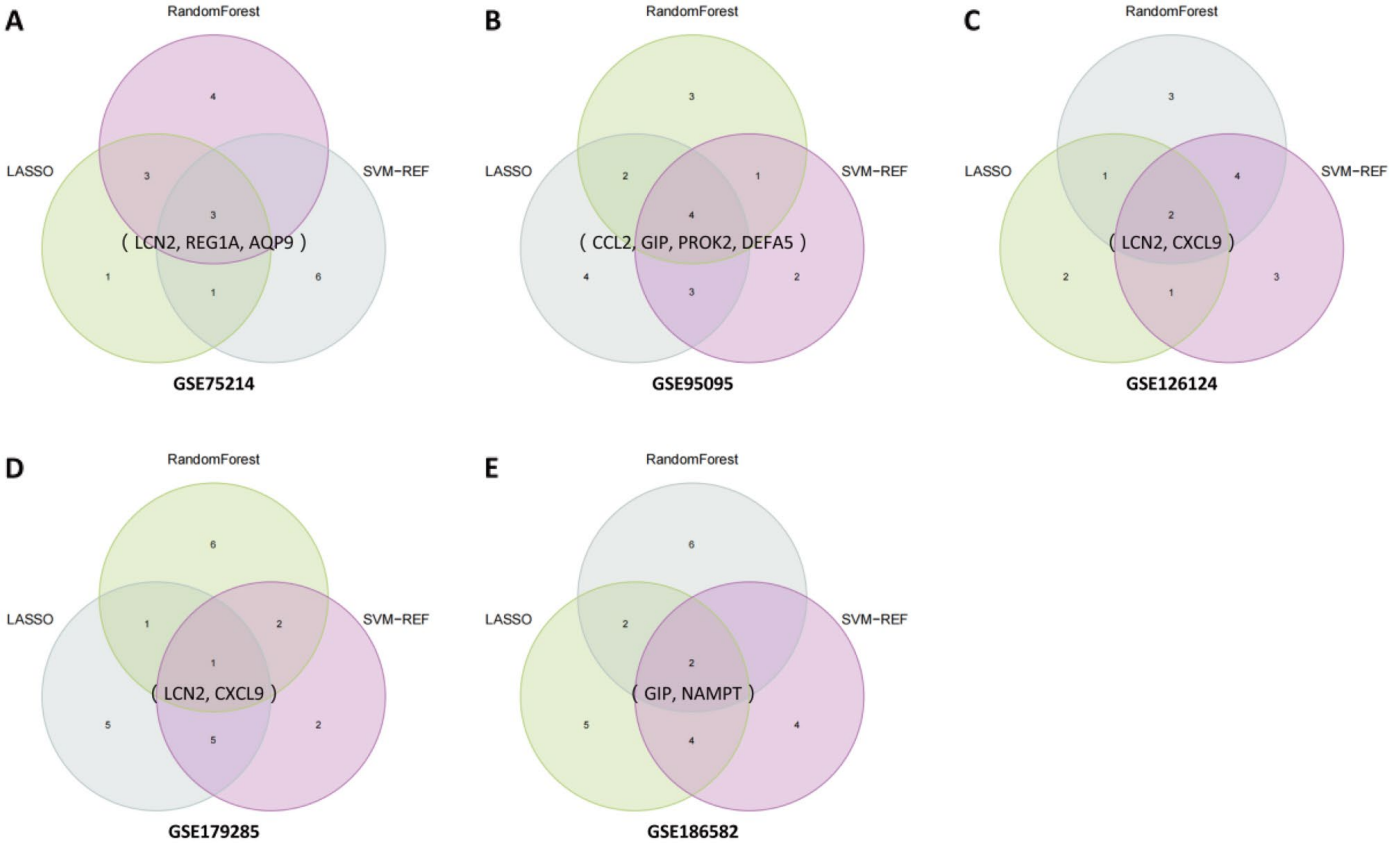
Determination of characteristic genes in GSE75214 (**A**), GSE95095 (**B**), GSE126124 (**C**), GSE179285 (**D**), and GSE186582 (**E**).

Characteristic genes were finally obtained by taking the intersection of the results of the three feature selection methods, as depicted in Fig. 5. The selected characteristic genes were LCN2, REG1A, and AQP9 for GSE75214; CCL2, GIP, PROK2, and DEFA5 for GSE95095; LCN2 and CXCL9 for GSE126124; LCN2 for GSE179285; and GIP and NAMPT for GSE186582.

### Verification of characteristic genes in RRA datasets and clinical samples

**Figure 6:**
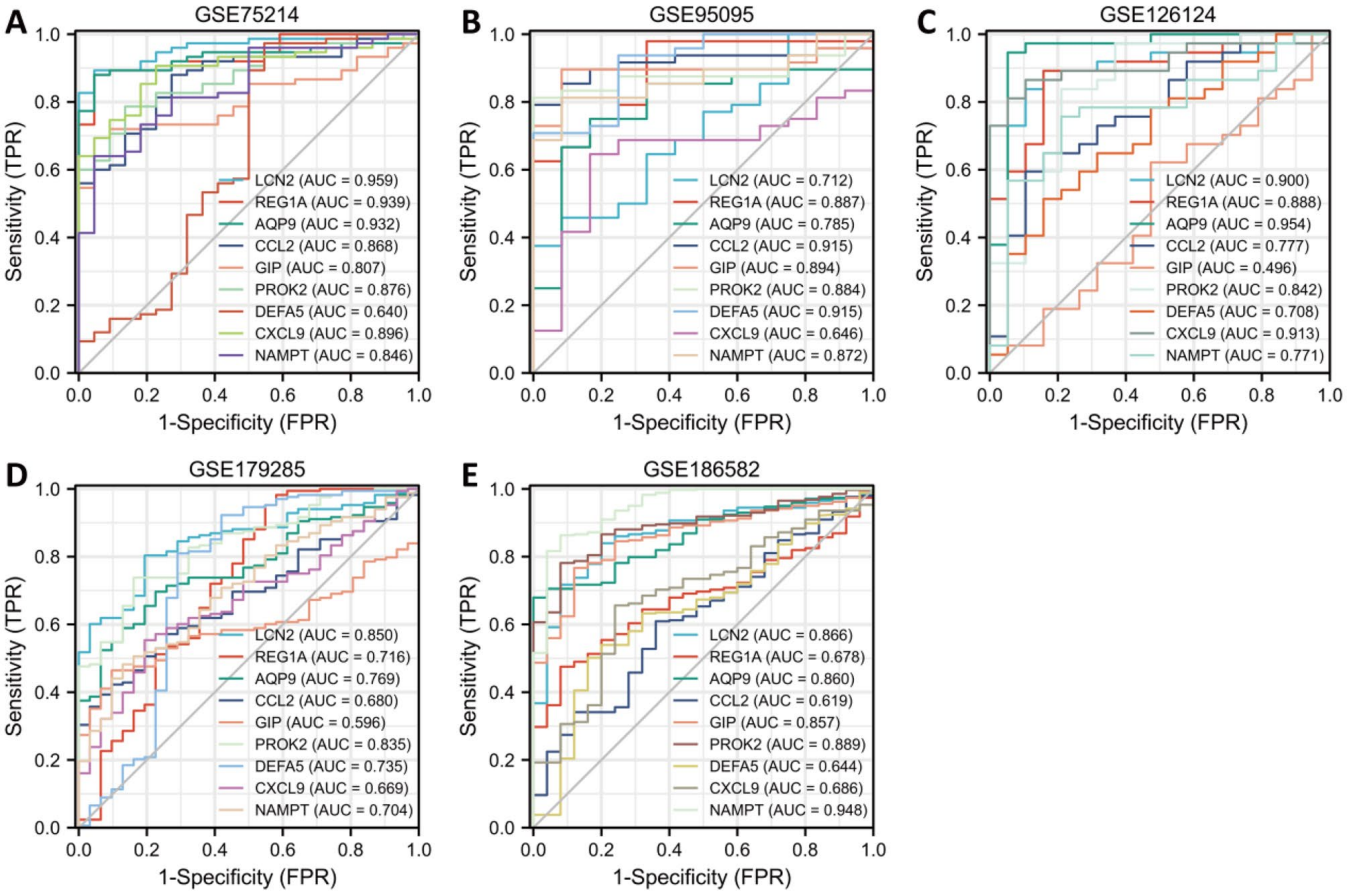
Diagnostic efficacy of characteristic genes in the prediction of CD progression and external verification of the expression of characteristic genes. ROC curves estimating the diagnostic performance of characteristic genes, including LCN2, REG1A, AQP9, CCL2, GIP, PROK2, DEFA5, CXCL9, and NAMPT, in the identification of CD patients in the GSE75214 (**A**), GSE95095 (**B**), GSE126124 (**C**), GSE179285 (**D**), and GSE186582 (**E**) datasets.

To further verify the value of the characteristic genes as diagnostic markers, we explored ROC curves in different cohorts included in the RRA analysis (Fig. 6). The results showed that the AUCs of AQP9 and PROK2 were greater than 0.75 and those of LCN2 and NAMPT were greater than 0.70 in all five cohorts included in the RRA. Therefore, these genes have good application value as biomarkers in CD diagnosis and treatment.

**Figure 7:**
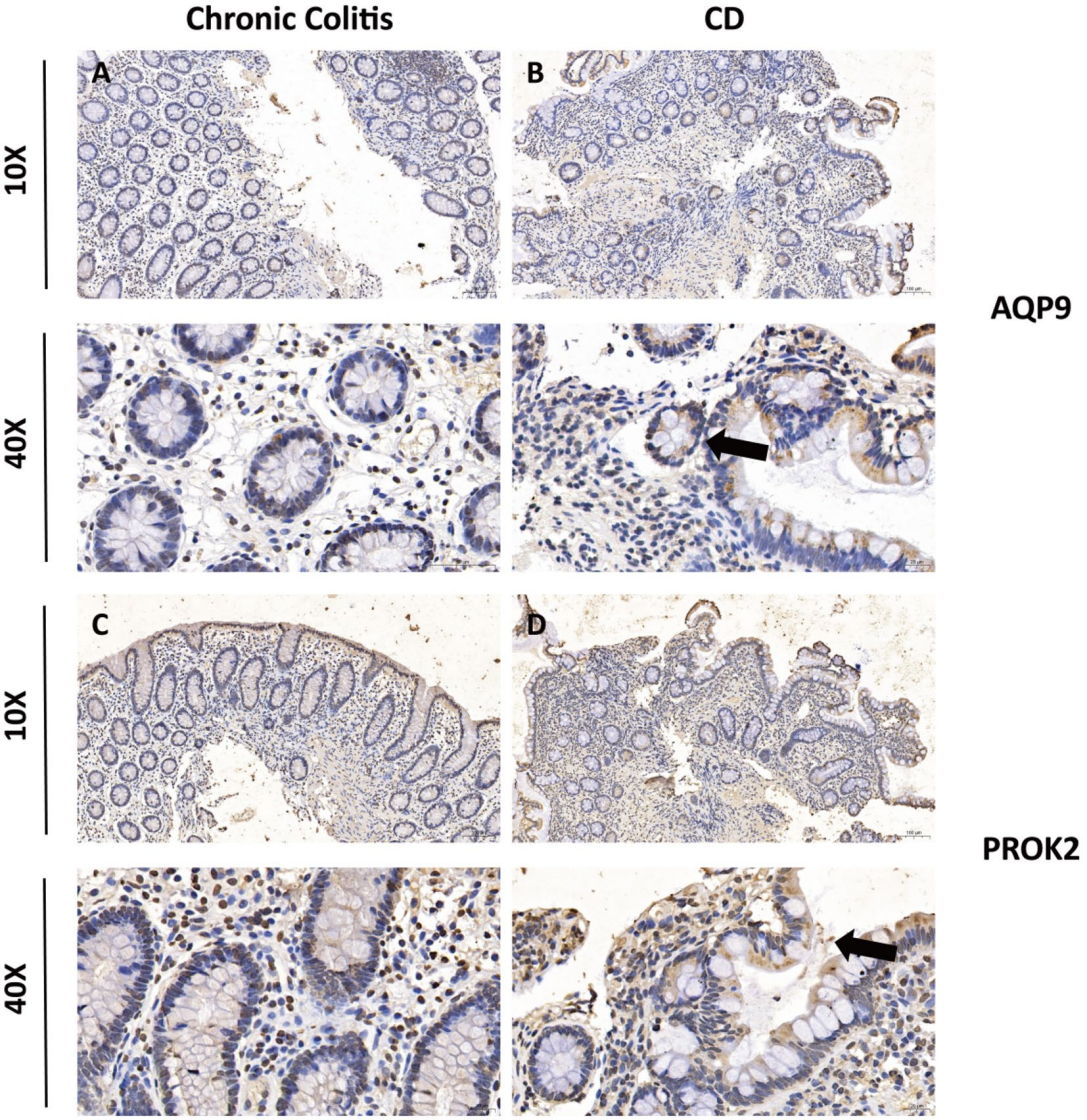
Validation of the expression of two prognostic genes in tissue samples. The IHC staining figures show representative images of the expression levels of AQP9 (**A**,**B**) and PROK2 (**C**,**D**) in chronic colitis and CD colon samples. The black arrow indicates the positive area of the IHC test.

To verify the accuracy of the results, chronic colitis and CD tissue specimens were examined by IHC for the expression levels of AQP9 and PROK2. Representative images of IHC staining are illustrated in Fig. 7A–D. We found that IHC expression of AQP9 and PROK2 in CD tissues was higher than that in chronic colitis tissues.

### Establishment of a machine learning score with 3 screened genes

**Figure 8:**
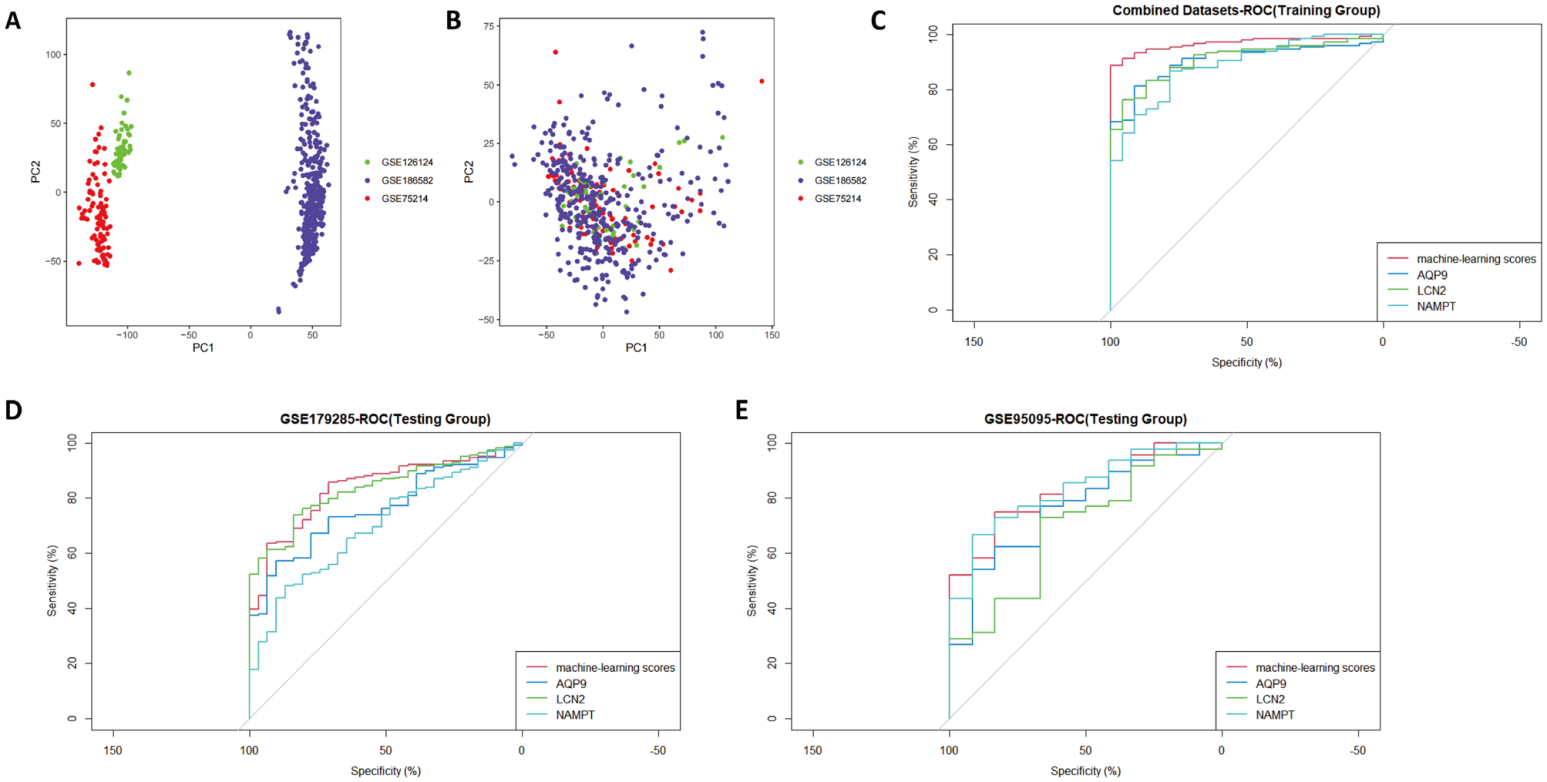
Establishment of a machine learning score with 3 screened genes. The GSE75214, GSE126124 and GSE186582 datasets were merged, and batch effects were further removed. PCA plots of different datasets are illustrated before (**A**) and after (**B**) batch effects were removed. To investigate the effects of the machine learning score, ROC curves were illustrated in the combined datasets (**C**) and GSE179285 (**D**) and GSE95095 (**E**).

To better explore the role of the four genes in the diagnosis of CD, we further constructed a machine learning score using machine learning scores for the four genes AQP9, LCN2, NAMPT, and PROK2. First, three datasets from the array platform of Affymetrix: GSE75214 (GPL6244. Affymetrix), GSE126124 (GPL6244, Affymetrix), and GSE186582 (GPL570, Affymetrix) were batch corrected and merged by the Combat function of sva packages, and the merged datasets were named Combined Datasets. We explored the batch differences of the 3 datasets before and after processing by PCA (Fig. 8A,B). The results showed that the batch differences were significantly reduced after Combat function treatment. We then performed univariable logistic regression analysis with the combined datasets as the training set and selected genes with P < 0.05 for multivariable logistic regression analysis (Table 2). The results showed that all four genes had P < 0.05 in the univariable logistic regression, and a total of three of these genes were included in the further multivariable regression analysis. The final machine learning score was defined as [Expression level of AQP9 × (2.644)] + [Expression level of LCN2 × (0.958)] + [Expression level of NAMPT × (1.115)].

**Table 2:** Univariable and multivariable logistic analysis for machine learning scores.

Genes	Univariable logistic regression						Multivariable logistic regression					
	B	SE	OR	CI	Z	P	B	SE	OR	CI	Z	P
AQP9	2.822	0.656	16.81	4.65–60.79	4.3	< 0.001	2.644	0.934	14.07	2.25–87.74	2.83	0.005
LCN2	0.928	0.17	2.53	1.81–3.53	5.445	< 0.001	0.958	0.262	2.61	1.56–4.36	3.662	< 0.001
NAMPT	2.235	0.49	9.34	3.58–24.42	4.563	< 0.001	1.115	0.556	3.05	1.03–9.06	2.004	0.045
PROK2	1.912	0.517	6.76	2.46–18.63	3.694	< 0.001	NA	NA	NA	NA	NA	NA

We then performed an ROC curve analysis and compared it with three single gene examinations, AQP9, LCN2, and NAMPT. The results showed an AUC of 0.969 for diagnosis using machine learning scores in the combined datasets (training group) (Fig. 8C), compared to 0.833 in GSE95095 (validation set 1) (Fig. 8D) and 0.838 in GSE179285 (validation set 2) (Fig. 8E). The diagnostic value of the scores was compared with other single-gene AQP9, LCN2, and NAMPT3, and the results showed good robustness of the machine learning scores across cohorts in multiple centers.

### Signaling pathways involving characteristic genes in the combined cohort

**Figure 9:**
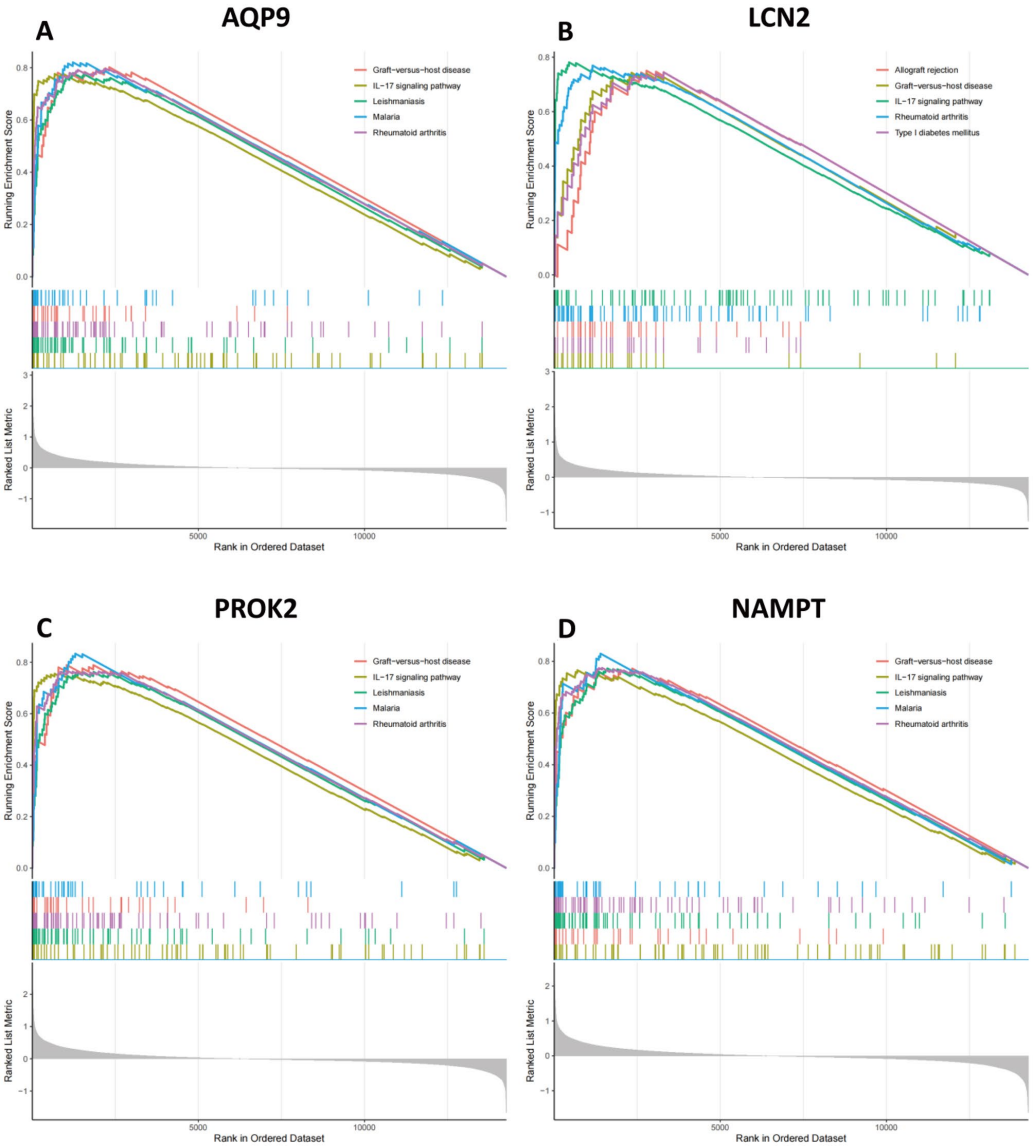
GSEA identifies signaling pathways involving characteristic genes. The main signaling pathways significantly enriched in highly expressed characteristic genes (**A**) AQP9, (**B**) LCN2, (**C**) PROK2, and (**D**) NAMPT.

The signaling pathways associated with the selected characteristic genes were evaluated using GSEA in the combined cohort (Fig. 9A–D). Our analysis indicated that AQP9, PROK2, LCN2, and NAMPT were positively linked to the IL-17/IL-17-associated signaling pathway (rheumatoid arthritis, systemic lupus erythematosus, and type 1 diabetes mellitus), immune-related disorders (allograft rejection and graft-versus-host disease) or infection (leishmaniasis and malaria)^8^.

### Analysis of correlation between related biomarkers and infiltrating immune cells

**Figure 10:**
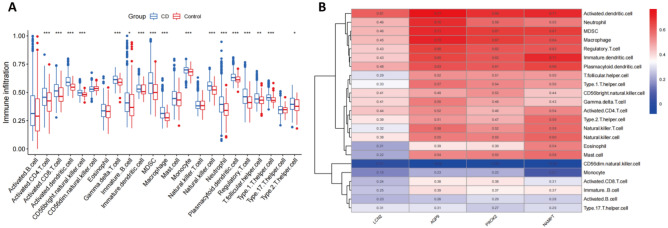
Immune cell infiltration analysis. (**A**) Level of immune infiltration between CD and normal samples. (**B**) Correlation heatmap showing the correlation between 23 kinds of infiltrating immune cells and 4 characteristic genes. Red denotes a positive correlation, blue denotes a negative correlation, and the size of the colored square denotes the correlation intensity.

By performing ssGSEA, we compared the infiltration levels of most immune cell populations between CD and normal samples in the combined cohort. The results showed several immune cell types, including dendritic cells, macrophages, regulatory T cells and NK cells, to be more abundant in CD patients than in controls in most of the datasets included in the RRA analysis (Fig. 10A). Further analysis showed that AQP9, PROK2, LCN2, and NAMPT correlated mainly positively with the differentially expressed cell types, indicating their value as biomarkers in CD (Fig. 10B).

## Discussion

CD is a chronic and relapsing inflammatory condition of the gastrointestinal tract that occurs following immune system dysregulation^35,36^. In this study, we integrated multiple datasets by RRA analysis to thoroughly identify DEGs and several immune-related genes via three machine learning methods. Currently, the pathogenesis of CD is still unclear, and relapse easily occurs after treatment^37^. Therefore, identifying novel intervention targets and investigating potential biomarkers as diagnostic indicators are essential to clarifying the cellular and molecular mechanisms contributing to CD pathogenesis^38^.

Our RRA analysis was conducted using five CD cohorts, identifying 203 DEGs. To the best of our knowledge, this study involves one of the largest sample sizes in current research on CD, including 671 cases of CD and 109 controls. The advantage of RRA analysis is that it can integrate different sequencing platforms and multicenter studies and effectively reduce batch differences to better identify DEGs^13^. In this study, most of the datasets published to date were retrieved and reviewed, and the retrieval strategies of the included and excluded datasets were clearly defined, which improves the credibility of the conclusions.

Analysis of PPI networks was performed for all DEGs, and MCODE was used to identify key functional gene modules^39^. Functional enrichment analysis revealed that the IL-17 signaling pathway plays an important role in the progression of CD for both DEGs and the top two modules with the highest score. IL-17 is the key cytokine produced by Th17 cells and has versatile functions^8^. Although Th1 and Th2 cells were once considered to be the only T-cell lineages resulting from progenitor CD4+ helper T cells, the discovery of Th17 cells as a distinct lineage of CD4+ helper T cells has changed our understanding of chronic inflammatory diseases such as CD and indicated a new way in which immune responses trigger intestinal tissue damage^8,40,41^. Studies have reported the role of IL-17 in a group of seemingly unrelated diseases that are triggered by or result from dysregulation of the innate and adaptive immune systems, including CD, UC, psoriasis^42,43^, rheumatoid arthritis^44–48^, and systemic lupus erythematosus^49–51^, among others. Interestingly, although overexpression of IL-17 in CD has been reported, its exact role in IBD is still controversial^8,40,52,53^. Nevertheless, similar to our results, high IL-17 mRNA expression levels have been detected in intestinal mucosa samples of patients with active CD as well as those with UC^54,55^. Mice deficient in IL-17 or treated with anti-IL-17 exhibit severe epithelial damage in the colon, indicating that IL-17 acts as a protective factor^56,57^. KEGG analysis also highlighted the role of the TNF pathway in CD. In the clinic, anti-TNF therapy was approved for use in CD in 1998, leading to increased rates of response and remission^58^. Our GO-BP analysis showed the DEGs and two modules to be mainly involved in inflammation. Notably, gene enrichment analysis showed the DEGs to mainly participate in the humoral immune response/antimicrobial humoral immune response. A widely accepted theory about the etiopathogenesis of CD is that the disorder is caused by an aggressive immune response to microorganisms of the intestinal microbiota in genetically predisposed individuals^59^. Immunoglobulin A (IgA) and immunoglobulin M (IgM) antibodies generated by the immune system are essential for maintaining mutualism between our bodies and the microbes that colonize our mucosal surfaces^60^. In addition, although intestinal IgG responses are limited in the healthy adult gut, several studies have demonstrated the importance of B cells and IgG in the pathogen containment and elimination effects that dominate those of IgA and IgM^61–66^.

We selected immune-related genes from among 203 DEGs. Traditionally, the multifactor logistic regression model is utilized for model building, the premise of which is that all included factors are independent of each other. However, this is certainly difficult to achieve for high-dimensional data such as array datasets, which may lead to overfitting of the model due to serious gene multicollinearity, resulting in reduced generalization ability of the model. Therefore, three machine learning methods, namely, LASSO, SVM-RFE, and RF, were applied to select characteristic genes, and the intersecting genes were further examined by ROC curve and IHC analyses. Four characteristic genes, AQP9, PROK2, LCN2, and NAMPT, exhibited superior diagnostic value in multiple cohorts.

Aquaporin 9 (AQP9) belongs to the aquaporin family of water-selective membrane channels that play a role in specialized leukocyte functions such as immunological response and bactericidal activity. Several studies have reported that AQP9 is a promising biomarker in CD patients^12,67,68^. Interestingly, AQP9 has also been reported as a prognostic indicator of many cancers in recent years and is related to immune infiltration^69–72^. Similar to CD, AQP9 expression is significantly increased in colon cancer^69^. In view of the correlation between long-term chronic IBD and AQP9, determining whether AQP9 plays a role in the transformation of IBD into cancer is a very valuable research direction. Although the mechanism of AQP9 in CD is not clear at present, it has been reported that AQP9 is required for inflammatory responses and DC maturation and that its expression level is markedly elevated by LPS exposure^73^. The inflammatory cytokine PROK2 (prokineticin 2) is produced primarily by macrophages and neutrophils invading sites of tissue damage, and increased levels of PROK2 have been reported in gut inflammation^74,75^. It remains to be determined exactly how elevated PROK2 causes visceral nociception. Several reports have shown that PROK2 released by inflammatory cells may cause inflammatory pain by attracting monocytes and macrophages as well as by stimulating the secretion of inflammatory and analgesic cytokines^76–81^.

LCN2 (lipocalin 2) belongs to the lipocalin family. LCN2 has been reported as a biomarker of IBD not only in the intestinal mucosa but also in feces^82–84^. In our research, LCN2 was selected using multiple CD samples in datasets including GSE75214, GSE126124 and GSE179285. Mechanistically, LCN2 is produced by a variety of cell types, including myeloid and intestinal epithelial cells, which seem to be particularly important in IBD. In IBD remission, persistent mucosal overexpression of LCN2 makes it a promising candidate for molecular inflammation that warrants investigation^85^. NAMPT (nicotinamide phosphoribosyltransferase) belongs to the nicotinic acid phosphoribosyltransferase (NAPRTase) family, and by catalyzing the rate-limiting step of NAD salvage, it is critical for maintenance of the cellular nicotinamide adenine dinucleotide (NAD) supply^86^. Considering that NAD is a major coenzyme in bioenergetic processes, NAMPT is biologically indispensable, and it has been implicated in a variety of inflammatory disorders, such as tumorigenesis, diabetes, rheumatoid arthritis and sepsis^87–90^. Moreover, NAMPT overexpression has been identified as a marker of severity in pediatric IBD^91,92^. A small molecule inhibitor, FK866, inhibits NAMPT enzymatic activity with little toxicity, making it a potentially useful drug for various inflammatory conditions^89,93,94^.

There are also some deficiencies in this study. (1) Although RRA analysis reduces batch differences in different study combinations, the inclusion criteria for cases, sample size and treatment received by the patients in these studies differ, which introduces bias in the final results. (2) Due to differences in gene probes between different technical platforms, some key genes may not be detected in a cohort, which results in a significant reduction in the number of candidate genes for analysis in the combined cohort, thus omitting some important biomarkers. (3) Larger-scale experimental validation is needed to prove the clinical value of these markers.

## Conclusion

In conclusion, our analysis reveals putative key biomarkers in CD, i.e., AQP9, PROK2, LCN2, and NAMPT. ssGSEA showed obviously elevated levels of DCs and innate immune cells, such as macrophages and NK cells, in CD, consistent with the gene enrichment results that the DEGs are mainly involved in the IL-17 signaling pathway and humoral immune response. Importantly, the identified biomarkers were validated by multiple external datasets and by IHC in independent clinical samples. Finally, the identified biomarkers correlate with elevated immune cell types, representing key features of the immune response, which might—in addition to serving as biomarkers for diagnostic purposes—prove to be efficient indicators of disease risk or improvement.

## Supplementary Information

Supplementary Figure 1.

Supplementary Table 1.

Supplementary Table 2.

Supplementary Table 3.

Supplementary Table 4.

Supplementary Table 5.

## Data Availability

The datasets analyzed during the current research are all available in Gene Expression Omnibus (http://www.ncbi.nlm.nih.gov/geo/). The data used to support the findings of this study are available from the corresponding author upon request.
