# Supplementary material for: Integrated multiple microarray studies by robust rank aggregation to identify immune-associated biomarkers in Crohn's disease based on three machine learning methods
Source: Sci Rep. 2023 Feb 15;13:2694. doi: 10.1038/s41598-022-26345-1 (PMC9931764; doi:10.1038/s41598-022-26345-1)
Supplement: Supplementary file 1 — Supplementary Figure 1. [file 41598_2022_26345_MOESM1_ESM.docx]

**
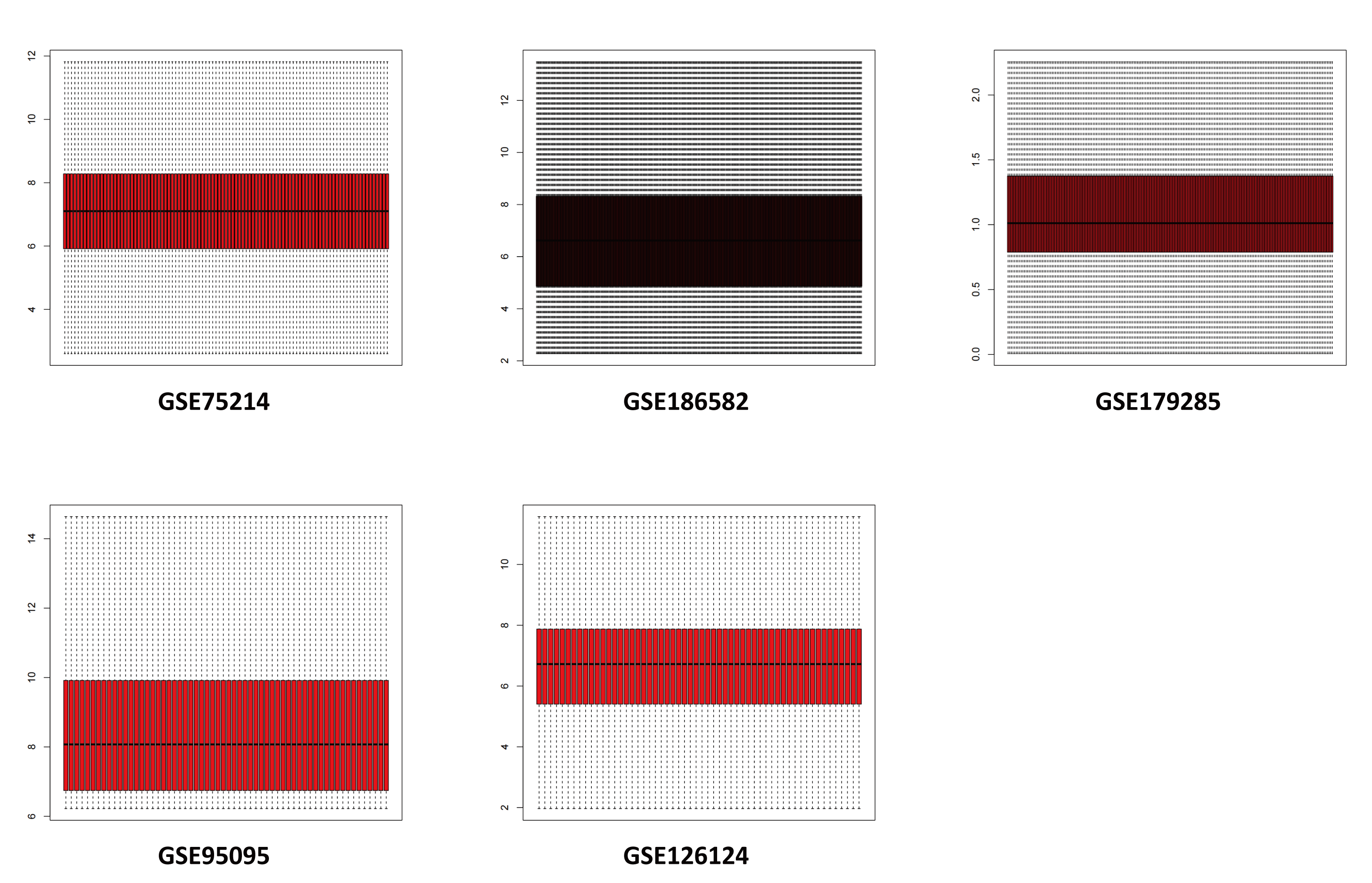

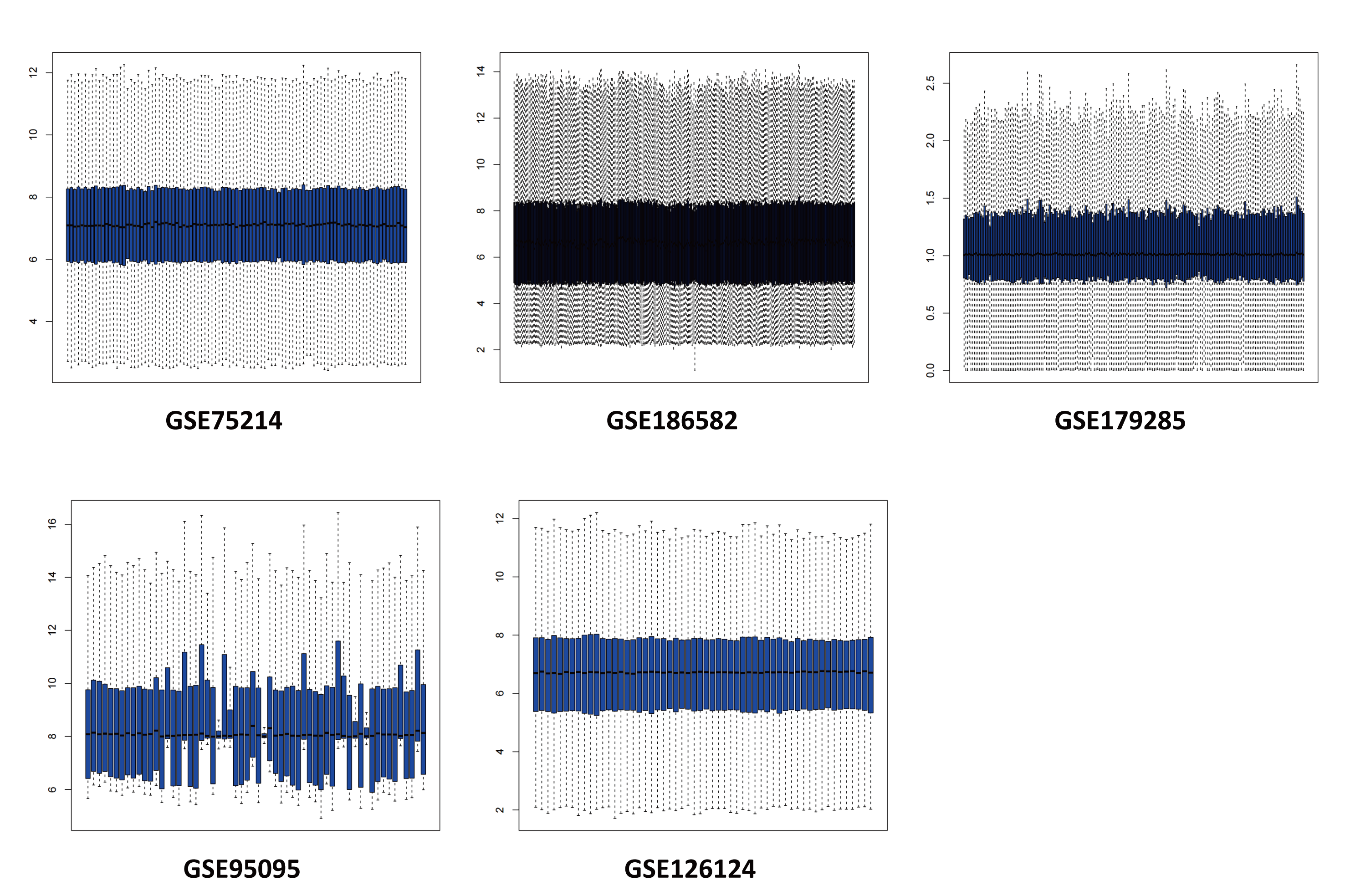
**

**Supplementary Figure 1：Standardization of gene expression**. Standardization of gene expression in GSE75214, GSE186582, GSE179285, GSE95095, GSE126124 datasets. The blue bar represents the data before normalization, and the red bar represents the data after normalization.
